# Phylogenetic Diversity of *Vibrio cholerae* Associated with Endemic Cholera in Mexico from 1991 to 2008

**DOI:** 10.1128/mBio.02160-15

**Published:** 2016-03-15

**Authors:** Seon Young Choi, Shah M. Rashed, Nur A. Hasan, Munirul Alam, Tarequl Islam, Abdus Sadique, Fatema-Tuz Johura, Mark Eppinger, Jacques Ravel, Anwar Huq, Alejandro Cravioto, Rita R. Colwell

**Affiliations:** a Maryland Pathogen Research Institute, University of Maryland, College Park, Maryland, USA; b CosmosID, Inc., Rockville, Maryland, USA; c Center of Bioinformatics and Computational Biology, University of Maryland Institute of Advanced Computer Studies, University of Maryland, College Park, Maryland, USA; d International Centre for Diarrhoeal Disease Research, Bangladesh (icddr,b), Dhaka, Bangladesh; e Department of Biology and South Texas Center for Emerging Infectious Diseases (STCEID), University of Texas at San Antonio, San Antonio, Texas, USA; f Institute for Genome Sciences (IGS), University of Maryland, School of Medicine, Baltimore, Maryland, USA; g Maryland Institute of Applied Environmental Health, University of Maryland, College Park, Maryland, USA; h Global Evaluative Sciences USA, Inc., Seattle, Washington, USA; i Johns Hopkins Bloomberg School of Public Health, Baltimore, Maryland, USA

## Abstract

An outbreak of cholera occurred in 1991 in Mexico, where it had not been reported for more than a century and is now endemic. *Vibrio cholerae* O1 prototype El Tor and classical strains coexist with altered El Tor strains (1991 to 1997). Nontoxigenic (CTX^−^) *V. cholerae* El Tor dominated toxigenic (CTX^+^) strains (2001 to 2003), but *V. cholerae* CTX^+^ variant El Tor was isolated during 2004 to 2008, outcompeting CTX^−^ *V. cholerae*. Genomes of six Mexican *V. cholerae* O1 strains isolated during 1991 to 2008 were sequenced and compared with both contemporary and archived strains of *V. cholerae*. Three were CTX^+^ El Tor, two were CTX^−^ El Tor, and the remaining strain was a CTX^+^ classical isolate. Whole-genome sequence analysis showed the six isolates belonged to five distinct phylogenetic clades. One CTX^−^ isolate is ancestral to the 6th and 7th pandemic CTX^+^ *V. cholerae* isolates. The other CTX^−^ isolate joined with CTX^−^ non-O1/O139 isolates from Haiti and seroconverted O1 isolates from Brazil and Amazonia. One CTX^+^ isolate was phylogenetically placed with the sixth pandemic classical clade and the *V. cholerae* O395 classical reference strain. Two CTX^+^ El Tor isolates possessing intact *Vibrio* seventh pandemic island II (VSP-II) are related to hybrid El Tor isolates from Mozambique and Bangladesh. The third CTX^+^ El Tor isolate contained West African-South American (WASA) recombination in VSP-II and showed relatedness to isolates from Peru and Brazil. Except for one isolate, all Mexican isolates lack SXT/R391 integrative conjugative elements (ICEs) and sensitivity to selected antibiotics, with one isolate resistant to streptomycin. No isolates were related to contemporary isolates from Asia, Africa, or Haiti, indicating phylogenetic diversity.

## IMPORTANCE

Sequencing of genomes of *V. cholerae* is critical if genetic changes occurring over time in the circulating population of an area of endemicity are to be understood. Although cholera outbreaks occurred rarely in Mexico prior to the 1990s, genetically diverse *V. cholerae* O1 strains were isolated between 1991 and 2008. Despite the lack of strong evidence, the notion that cholera was transmitted from Africa to Latin America has been proposed in the literature. In this study, we have applied whole-genome sequence analysis to a set of 124 *V. cholerae* strains, including six Mexican isolates, to determine their phylogenetic relationships. Phylogenetic analysis indicated the six *V. cholerae* O1 isolates belong to five phylogenetic clades: i.e., basal, nontoxigenic, classical, El Tor, and hybrid El Tor. Thus, the results of phylogenetic analysis, coupled with CTXϕ array and antibiotic susceptibility, do not support single-source transmission of cholera to Mexico from African countries. The association of indigenous populations of *V. cholerae* that has been observed in this study suggests it plays a significant role in the dynamics of cholera in Mexico.

## INTRODUCTION

Cholera, a deadly waterborne disease, is caused by *Vibrio cholerae* and continues to be a health hazard for millions around the world, particularly in developing countries. Of more than 200 “O” serogroups, only *V. cholerae* O1 and O139 have been associated with cholera epidemics. Serogroup O1 has been classified into two biotypes, classical (CL) and El Tor (ET), the latter linked to the ongoing 7th pandemic first reported in 1961 (1, 2). Although cholera has been endemic in the Ganges Delta region of South Asia for centuries, several countries of sub-Saharan Africa and Latin America were severely affected during the 7th pandemic and subsequently are now considered areas of endemicity (1). That is, cholera appeared in Mexico in June 1991, after the Latin American epidemic had begun along the Peruvian coast in January 1991 (3). The disease soon broke out in neighboring countries by 1992, with the exceptions of Uruguay and French Guyana (4). In Mexico, a total of 43,536 cholera cases were reported between 1991 and 1996, with a substantial number of deaths (3). Epidemiological investigations confirmed the association of *V. cholerae* O1 biotype El Tor with the majority of those cholera cases, although the classical biotype was isolated from some cases in Mexico during subsequent years until 1997 (4–7).

It has long been established that *V. cholerae* O1 had caused seven pandemics since 1817, of which the 7th pandemic is the largest, considering its longevity and geographical distribution. *V. cholerae* El Tor replaced the classical biotype of the 6th pandemic and presumably earlier pandemics (1, 8). Variants of El Tor (hybrid El Tor and/or altered El Tor) possessing classical biotype-specific traits have been reported in Asia, Africa, and Latin America (5, 9, 10). Genetic changes (i.e., gain or loss of mobile genetic elements and genomic islands) occur in *V. cholerae* due to its genomic plasticity (11). An example is the emergence of *V. cholerae* O139 in late 1992 in India, which is a non-O1 serogroup that caused a massive outbreak in South Asia and beyond (12, 13). Since 2001, variants of El Tor have been associated with cholera epidemics globally, including the recent epidemic in Haiti and previously Zimbabwe (14–16). Although significant advances have been made in the understanding of the genetics, epidemiology, and ecology of *V. cholerae* over the past two decades, the lack of an extensive genomic database severely limits source attribution for some of the recent outbreaks.

The cholera epidemic in Latin America was hypothesized to have been imported from areas of endemicity since Latin America had not reported cholera for more than 100 years prior to 1991 (17). Three hypotheses have been offered: (i) international trade ships from Asia discharged the pathogen into Peruvian ports in ballast water (18), (ii) immigrants who came from Africa to Latin America in the 1970s brought the pathogen with them (6, 19), and (iii) environmental factors (e.g., El Nino) played a significant role (20, 21). Preliminary analysis using molecular typing indicated *V. cholerae* strains isolated in Latin America during the 1990s’ epidemic were clonal and represented intrusion of the seventh pandemic El Tor strain into the Western hemisphere (unrelated to the U.S. Gulf Coast clone) (6). However, subsequent genomic analysis of 30 single-nucleotide polymorphisms (SNPs) indicated close relatedness of the Latin American isolates from the 1990s to African strains isolated in the 1970s and 1990s (19). This finding was supported by a recent phylogenetic analysis showing isolates from the Latin American epidemic in the 1990s were related to a *V. cholerae* strain from Angola, the study that analyzed only seventh pandemic El Tor strains from the Latin American epidemic that carried the *ctxB*3 genotype (*B3* allele) (8). However, *V. cholerae* altered El Tor has been found to coexist with classical and prototype El Tor in Mexico since the Latin American epidemic began (5). A serious limitation of that retrospective epidemiological study was that the analysis included only a limited number of strains collected spatio-temporally, thereby masking the full genetic diversity of the Mexican *V. cholerae* population. Phenotypic and genotypic characteristics of 182 *V. cholerae* O1 strains from Mexico that had been isolated between 1983 and 2008 previously had been reported to have several unique features (5, 7, 22) (see Table S1 in the supplemental material). In this study, six *V. cholerae* O1 isolates from Mexico were selected (Table 1) based on previously published data (5, 7, 22), for whole-genome sequencing to compare their genomes with genomes of 124 *V. cholerae* archival and recent isolates to elucidate the evolutionary dynamics of *V. cholerae* in Mexico.

**TABLE 1:** Characteristics of *Vibrio cholerae* serogroup O1 strains analyzed in this study^a^

Strain	Serotype	Biotype	Source	Yr of isolation	CTXΦ	Accession no.
CP1032	Ogawa	El Tor	Human	1991	+	ALDA00000000
95412	Inaba	Classical	Human	1997	+	APFM00000000
CP1033	Ogawa	El Tor	Human	2000	+	AJRL00000000
CP1037	Ogawa	El Tor	Environment	2003	−	ALDB00000000
CP1035	Ogawa	El Tor	Human	2004	−	AJRM00000000
CP1030	Inaba	El Tor	Environment	2008	+	ALCZ00000000

a Mexico was the country of origin for all strains shown here.

## RESULTS AND DISCUSSION

### Variations in CTXΦ-RS1.

Four of the six isolates of *V. cholerae* O1 (95412, CP1030, CP1032, and CP1033) were lysogenic CTXΦ positive, while the remaining two isolates (CP1035 and CP1037) lacked CTXΦ (Table 1). Lysogenic CTXΦ contains two gene clusters, a core region and and RS2 element (23, 24). The core region comprises *ctxAB*, encoding cholera toxin (CT), and five other genes, namely, *psh*, *cep*, *orfU*, *ace*, and *zot*, that are required for phage morphogenesis. The RS2 element encodes proteins associated with CTXΦ replication (RstA), integration (RstB), and regulation (RstR) (23, 24). Satellite phage RS1 carries an additional *rstC* gene (encoding anti-repressor protein), along with the entire RS2 element that is usually present in the flanking region of CTXΦ in *V. cholerae* El Tor (24). The chromosomal location of CTXΦ and its orientation and copies of CTXΦ may differ among toxigenic *V. cholerae* strains (25–27). The CTXΦ-RS1 array of CP1030 has been shown to be unique, lacking RS1 and carrying a truncated CTXΦ instead of RS1 in the upstream region of CTXΦ (*B3* allele) in the large chromosome (Chr I) (7). The *V. cholerae* O1 El Tor strains isolated in Mexico between 2004 and 2008, show the same CTXΦ array (TLC-truncated CTX-CTXΦ*^B3^*) (7). Moreover, predicted CTXΦ mapping of El Tor isolates associated with the 1990s’ Latin American epidemic in Peru, Mexico, Bolivia, Columbia, and Argentina showed two copies of CTXΦ (*B3* allele) together with TLC and RS1 in Chr I (TLC-CTXΦ*^B3^*-CTXΦ*^B3^*-RS1) (8). CTXΦ arrays, either TLC-truncated CTX-CTXΦ*^B3^*, or TLC-CTXΦ*^B3^*-CTXΦ*^B3^*-RS1 detected in Latin American isolates was not found in El Tor, altered El Tor, or El Tor variants from Asia, Africa, and Haiti that have been studied to date (8, 25–27). However, an isolate from Sweden was found to contain the latter. Recently, genomic analysis of *V. cholerae* O1 showed close relatedness between isolates from Latin America and Angola, but the CTXΦ array was different (8).

As shown in Table 2, the *rstA* and *rstB* gene sequence of *V. cholerae* 95412 classical is identical to that of the reference *V. cholerae* O395 classical isolate, whereas variation was observed in *V. cholerae* CTX^+^ El Tor isolates. *V. cholerae* CP1030, CP1032, and CP1033 contained three unique base substitutions in the *rstA* gene at 927 (T→C), 933 (C→T), and 942 (G→T), compared to *V. cholerae* N16961, CIRS101, and the recent Haitian isolate HCO1. In addition, CP1032 had a base substitution at 315 (T→C) in the *rstA* gene. Interestingly, all point mutations are synonymous for RstA. DNA sequence analysis of CP1030, CP1032, and CP1033 at the *rstB* gene showed a GTA deletion at positions 77 to 79 and polymorphism at positions 90 (A→T), 96 (T→C), 108 (G→A), and 192 (A→G), unlike *V. cholerae* El Tor strains, except or the GTA deletion, which had been reported in Haitian isolates (14).

**TABLE 2:** Sites of nucleotide polymorphisms in CTX prophages

Strain	Country of origin	Yr of isolation	Gene position	*rstR* type	*rstA* polymorphism at position:														*rstB* polymorphism at position:								No. of copies of heptamer in *zot-ctxA* region^a^	*ctxB* allele **type
					27	162	183	258	315	345	516	540	579	609	774	927	933	942	77–79	90	96	108	192	288	291	363		
N16961	Bangladesh	1975	CTX^ET^	ET	C	C	C	G	T	G	G	A	T	T	C	T	C	G	GTA	A	T	G	A	A	C	A	4	*B3*
O395	India	1965	CTX^CL^	CL	T	T	A	C	*	T	A	G	C	C	T	*	*	*	−^c^	T	C	*	*	G	T	*	7	*B1*
CIRS101	Bangladesh	2002	CTX^HYB^	ET	*^b^	*	*	*	*	*	*	*	*	*	*	*	*	*	*	*	*	*	*	*	*	*	3	*B1*
HCO1	Haiti	2010	CTX^HYB^	ET	*	*	*	*	*	*	*	*	*	*	*	*	*	*	−	*	*	*	*	*	*	*	5	*B1*
95412	Mexico	1997	CTX^CL^	CL	T	T	A	C	*	T	A	G	C	C	T	*	*	*	−	T	C	*	*	G	T	*	6	*B1*
CP1030	Mexico	2008	CTX^ET^	ET	*	*	*	*	*	*	*	*	*	*	*	C	T	T	−	T	C	A	*	*	*	*	4	*B3*
CP1032	Mexico	1991	CTX^HYB^	ET and CL	*	*	*	*	C	*	*	*	*	*	*	C	T	T	−	T	C	A	*	*	*	*	4	*B1*
CP1033	Mexico	2000	CTX^HYB^	ET and CL	*	*	*	*	*	*	*	*	*	*	*	C	T	T	−	T	C	A	G	*	*	G	4	*B1*

a Shown are the numbers of copies of the TTTTGAT heptamer repeat sequence.

b *, indicates sequence identical to that of *V. cholerae* N16961.

c −, GTA deletion.

Virulence gene expression in *V. cholerae* is regulated by ToxR, a transcriptional regulator that binds with the promoter region (between *zot* and *ctxA*) located upstream of *ctxAB*. The heptamer repeat sequences (TTTTGAT) directly influence the affinity of ToxR binding and promote binding of ToxR, which is followed by activation of the *ctxAB* promoter (28). As shown in Table 2, *V. cholerae* CP1030, CP1032, and CP1033 contain four copies of the heptamer repeat, like El Tor, altered El Tor, and hybrid variants from Asia and Africa. However, they differ from the Haitian isolates in having five repeats (14, 29). The *V. cholerae* 95412 classical isolate contains six copies of the heptamer repeat, unlike the classical *V. cholerae* reference strain O395, which possesses seven copies of the repeat (Table 2).

### *Vibrio* pathogenicity islands 1 and 2.

**FIG 1:**
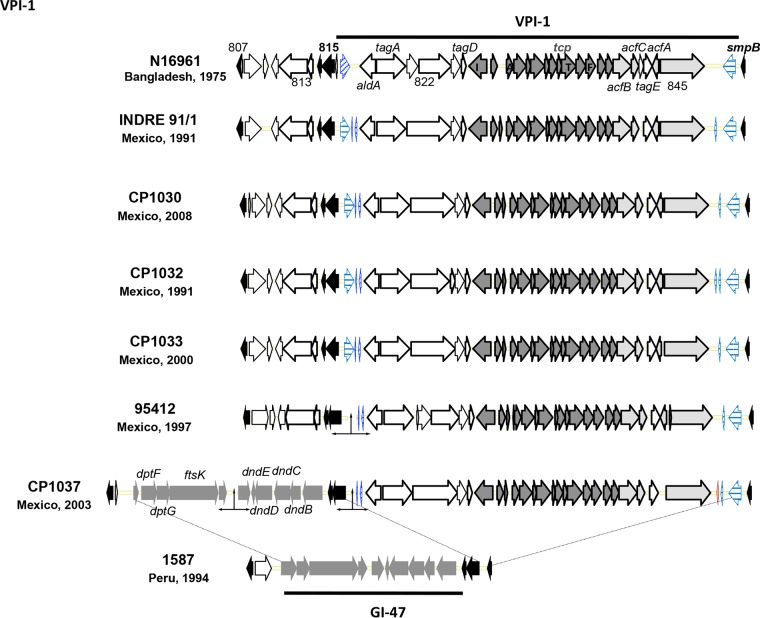
*Vibrio* pathogenicity island 1 (VPI-1) of *V. cholerae* O1 strains isolated in Mexico and reference El Tor strain N16961. Mexican CTX^−^ *V. cholerae* O1 strain CP1037 contains GI-47 in the upstream region of VPI-1.

*Vibrio* pathogenicity island-1 (VPI-1) encodes the toxin-coregulated pilus (TCP) that promotes colonization of intestinal mucosal epithelium, is involved in biofilm formation, and serves as the receptor for the lysogenic CTXΦ (30). Five of the six *V. cholerae* O1 isolates from Mexico contained VPI-1, but CP1035 lacked this gene cluster. As shown in Fig. 1, *V. cholerae* CP1030, CP1032, and CP1033 possess VPI-1 of the seventh pandemic *V. cholerae* El Tor isolates, whereas the genetic organization of VPI-1of CP1037 is homologous to that of *V. cholerae* 95412 (classical), despite having a genomic island, GI-47, in the upstream region**.** Interestingly, the *tcpA* gene, encoding the major pilin subunit (TcpA) of CP1037, is different from the classical and El Tor *tcpA* genes. The TCP region showed highest level of sequence polymorphism in VPI-1, with *tcpA* having the most divergence (31). Previous studies reported TcpA had significant differences in the epitope or antigenic structure when classical and El Tor biotype strains were compared (32). Four of the *V. cholerae* O1 isolates, CP1030, CP1032, CP1033, and 95412, contain the complete VPI-2, whereas the other isolates lack VPI-2. VPI-2 comprises several genes, including those encoding sialidase, the type I restriction modification system, and Mu-like prophage protein genes.

### *Vibrio* seventh pandemic islands.

**FIG 2:**
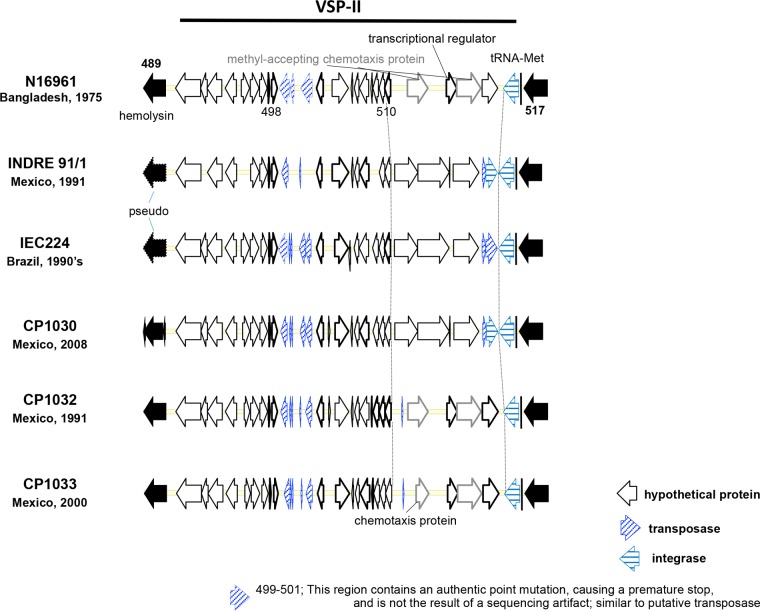
*Vibrio* seventh pandemic island II (VSP-II) of *V. cholerae* O1 strains isolated in Mexico and Brazil. VSP-II of hybrid *V. cholerae* O1 El Tor (CP1032 and CP1033) was similar to that of reference El Tor N16961. However, it is different from those of prototype El Tor isolates (CP1030, ICE224, and INDRE 91/1) in Mexico and Brazil.

The *Vibrio* seventh pandemic islands I and II (VSP-I and -II) in *V. cholerae* are characteristically found in El Tor strains, and they serve as a distinguishing marker from classical strains (33). However, a variant of the VSP-II gene cluster has also been detected in *V. cholerae* non-O1/O139 strains and in *Vibrio mimicus* (34, 35). *V. cholerae* El Tor strains CP1032 and CP1033 from Mexico contained all of the open reading frames (ORFs) in VSP-I and -II, whereas the CTX^−^ isolates CP1035 and CP1037 lack VSP-I and -II, as does the classical strain 95412. CP1030 possesses a variant VSP-II with an insertion between VC0510 and VC0516 (Fig. 2), commonly referred to as the West African and South American (WASA) insertion (8). An identical VSP-II gene cluster has been reported in *V. cholerae* isolated in Peru and Angola (36, 37). Conversely, the VSP-II gene cluster in contemporary *V. cholerae* isolates from Asia and Haiti has a 14.4-kb deletion that spans the ORF from VC0495 to VC0512 (CIRS101 type VSP-II) (14, 35, 38). The distribution of the variant VSP-II types among the *V. cholerae* isolates suggests this island contains hot spots highly prone to genetic rearrangement by recombination (35).

### GIs and ICEs.

*V. cholerae* O1 isolates from Mexico contain diverse genomic islands (GIs) that differ among the El Tor, classical, and CTX^−^ strains (see Table S2 in the supplemental material). *V. cholerae* El Tor isolates CP1030, CP1032, and CP1033 uniformly contained GI-1 to GI-10 and GI-85. *V. cholerae* CP1033 (14), on the other hand, contains GI-15 in the large chromosome, which encodes the putative integrase found in the Mozambique variant of *V. cholerae* (B-33) and also in hybrid isolates of CP1067 from Bangladesh, that had been isolated in 1991. Moreover, *V. cholerae* CP1030 contains the WASA1 genomic island, which has been reported previously in West African and South American strains (8). *V. cholerae* classical strain 95412 has GIs typical of the reference classical strain O395, along with GI-11 and GI-21 in the small chromosome (see Table S2). GI-11 encodes the kappa prophage, whereas the function of GI-21 (~34 kb) has not yet been identified. *V. cholerae* CP1035 contains genomic islands that are similar to those of *V. cholerae* non-O1/O139 and differ from classical and El Tor strains. CP1035 contains several previously described genomic islands, including GI-125 and GI-126, encoding a type I restriction modification system and integrase. Interestingly, CP1037 carries GI-36, which has been detected previously in *V. cholerae* non-O1/O139 TM11079-80 and Amazonia, isolated in Brazil. CP1037 also possesses GI-47 in the upstream region of VPI-1, as previously observed in Peruvian *V. cholerae* isolated in 1994 (Fig. 1) and a unique genomic island, GI-112, carrying *umuCD* and a nucleotidyltransferase gene (see Table S2) (11, 14).

The integrating and conjugative elements (ICEs) are self-transmissible mobile genetic elements in bacteria that confer resistance to various antibiotics. SXT is an ~100-kb ICE originally discovered in *V. cholerae* O139 (39). Since the emergence of *V. cholerae* O139 on the Indian subcontinent in 1992, the SXT/R391 ICE has been reported to be present in most clinical *V. cholerae* O1 or O139 strains isolated in Asia and Africa (8). *V. cholerae* isolates carrying the SXT/R391 ICE are resistant to streptomycin, chloramphenicol, sulfamethoxazole, and trimethoprim (39). Results of recent phylogenetic analysis suggest *V. cholerae* O1 acquired SXT/R391 ICE sometime between 1978 and 1984, before its discovery in *V. cholerae* O139, and it is hypothesized that it provides a selective advantage to *V. cholerae* O1, allowing it to be globally disseminated (8). In the present study, except for CP1035, all of the Mexican isolates lacked the SXT/R391 ICE. The genome sequences of Latin American isolates (INDRE 91/1 [Mexico]; CP1044, CP1046, and CP1047 [Peru]; and IEC224, RC144, and 116059 [Brazil]) are devoid of SXT/R391 ICE. This observation was confirmed by PCR—i.e., except for CP1035, none of the Mexican isolates amplified DNA fragments for primers targeting the SXT integrase gene (*intSXT*) (40). Lack of the SXT/R391 ICE in epidemic strains isolated in Latin America in the 1990s has been reported (8). Absence of the SXT/R391 ICE among *V. cholerae* isolates has also been reported in a recent cholera outbreak in the Philippines (41). Antibiotic susceptibility analyses of the five Mexican isolates, CP1030, CP1032, CP1035, CP1037, and 95412, revealed all were sensitive to penicillin, ampicillin, streptomycin, chloramphenicol, trimethoprim-sulfamethoxazole, tetracycline, kanamycin, erythromycin, nalidixic acid, and ciprofloxacin. *V. cholerae* CP1033 shows resistance only to streptomycin. Despite possessing SXT/R391 ICE, CP1035 was sensitive to antibiotics, suggesting SXT/R391 ICE lacks genes conferring resistance to streptomycin, chloramphenicol, and trimethoprim-sulfamethoxazole*.* However, *V. cholerae* O1 strains showed resistance to different antibiotics in Asia and Africa at least a decade earlier than the 1990s’ Latin American epidemic. *V. cholerae* El Tor strains isolated in 1977 in Africa were resistant to multiple drugs, including tetracycline (42), and classical strains from Bangladesh isolated during 1982 to 1989 were resistant to ampicillin, furazolidone, and trimethoprim-sulfamethoxazole (22).

### LPS coding region.

**FIG 3:**
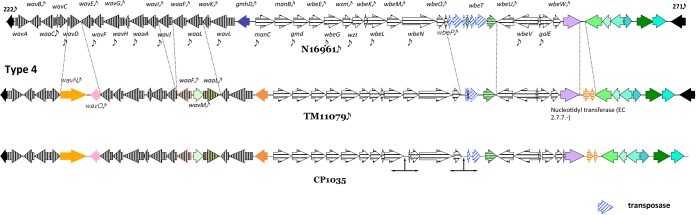
O antigen biosynthetic genes of *V. cholerae* O1 strains CP1035, TM11079, and N16961. The *wav* and wb* gene clusters of CP1035 are homologous to those of TM11079 and different from those of reference El Tor N16961.

The lipopolysaccharide (LPS) of *V. cholerae* is comprised of three main regions: lipid A, the core oligosaccharide (OS), and the O antigen. *V. cholerae* synthesizes the core OS and O antigen using the *wav* and wb* gene clusters, respectively (43). The *wav* gene cluster (VC0223 to -240) of the Mexican isolates is similar to that of *V. cholerae* N16961, except for CP1035, which is different in seven of the ORFs (Fig. 3). *V. cholerae* CP1035 has a *wav* gene cluster homologous to *V. cholerae* TM11079-80, an environmental strain isolated in Brazil in 1980 (Fig. 3). Interestingly, both strains are phenotypically El Tor, but they lack two major virulence-associated genomic islands, CTXΦ encoding CtxAB and *Vibrio* pathogenicity island VPI-1, which contains the genes for biosynthesis of toxin-coregulated pilus (TCP).

### Phylogenetics of the Mexican isolates.

**FIG 4:**
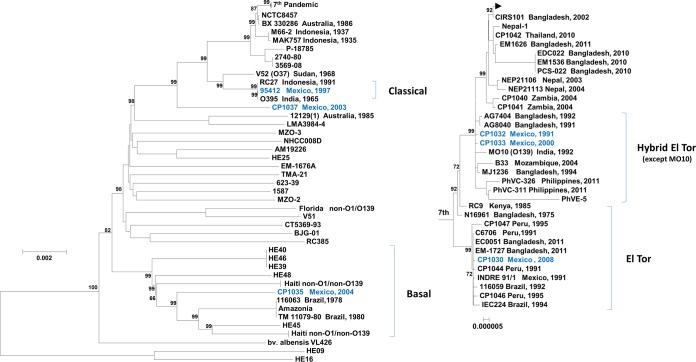
Neighbor-joining trees showing phylogenetic relationships of 124 *V. cholerae* genomes based on 905 orthologs of protein-coding genes (~897,461 bp). The two *V. cholerae* non-O1/O139 strains (HE09 and HE16) isolated from surface water during the 2010 cholera epidemic in Haiti were used as an outgroup of the tree, and bootstrap values are percentages of 1,000 replications. Mexican *V. cholerae* O1 strains are shown in blue, indicating the distribution among five distinct phylogenetic clades. The top node represents the genomes of isolates from Haiti, Bangladesh, Nepal, the United States, Cameroon, South Africa, the Russian Federation, Zimbabwe, and the Dominican Republic obtained between 2005 and 2011.

The phylogeny of the *V. cholerae* strains isolated in Mexico was determined by constructing a genome-relatedness neighbor-joining tree using homologous alignment of 905 orthologous protein-coding genes (~897,461 bp) of 124 *V. cholerae* genomes (Fig. 4), which placed El Tor, classical, and nontoxigenic *V. cholerae* isolates from Mexico into distinct phylogenetic clades. CP1035, a CTX^−^ isolate, was placed into a basal clade with other nontoxigenic non-O1/O139 isolates from Haiti and O1 isolates from Brazil and Amazonia. The other CTX^−^ isolate, *V. cholerae* CP1037, was phylogenetically placed into an independent node ancestral to all sixth and seventh pandemic isolates. The presence of ancestral isolate in the Latin American region is indicative of greater phylogenetic diversity and succession of indigenous *V. cholerae* populations in that ecosystem. The classical biotype isolate of *V. cholerae* 95412, isolated from Mexico in 1997, was placed into a monophyletic clade with the other sixth pandemic reference *V. cholerae* strain, O395, and RC27. Classical biotype strains are considered to have been outcompeted by seventh pandemic *V. cholerae* El Tor strains in the 1980s and have not been isolated in Asia and Africa after 1990 (22). In contrast, *V. cholerae* classical strains had been isolated in Mexico until 1997, even though *V. cholerae* El Tor strains were dominant at the beginning of the Latin American epidemic and during the years following, indicating the Mexican ecosystem to be a reservoir for the classical biotype of *V. cholerae* (5).

*V. cholerae* strains CP1032 and CP1033 isolated in Mexico were placed into the paraphyletic hybrid El Tor clade along with Mozambique and Matlab variants of *V. cholerae* El Tor, namely, B-33 and MJ-1236, together with *V. cholerae* O139 isolate MO10 (Fig. 4). These isolates also showed close relatedness to 1991 hybrid *V. cholerae* El Tor strain AG8040 isolated from patients in Bangladesh. Phylogenetic analysis of *V. cholerae* hybrid strains isolated in Mexico clearly shows a separation from contemporary *V. cholerae* El Tor and altered El Tor strains from Asia, Africa, and Haiti. The Matlab variant strains, isolated in 1994 in Bangladesh, were the first to have been reported in the literature as “hybrid,” showing classical biotype specific traits in an El Tor genetic background (9). A decade later, genetically similar hybrid variants were isolated in Mozambique during the 2004 cholera outbreak (11, 44). Isolation of *V. cholerae* CP1032 in 1991 in Mexico suggests hybrid El Tor *V. cholerae* was present at the same time in two different continents—i.e., Asia and America. *V. cholerae* CP1030 also belongs to the seventh pandemic clade. However, it clustered tightly into the monophyletic El Tor clade with *V. cholerae* strains isolated in Mexico, Peru, and Brazil during the Latin American epidemics of the 1990s but distant from recent isolates from Bangladesh, India, Nepal, and Thailand. Furthermore, Zambia, Zimbabwe, and Haiti isolates are also separated from CP1030, suggesting a conserved *V. cholerae* O1 clone that carries a truncated CTXΦ instead of RS1 in the upstream region of CTXΦ, circulating in the Mexican ecosystem during 2004 to 2008. Since 2000, variants of *V. cholerae* O1 El Tor have prevailed in areas of Asia and Africa where cholera is endemic, with *V. cholerae* prototype El Tor strains rarely isolated (45).

### Conclusion.

This study provides important insights into the molecular epidemiology of cholera in Mexico. Overall, the results of our study and previous studies show the existence of genetically diverse *V. cholerae* O1 in Mexico during 1991 to 2008 (5, 7). Considering the global epidemiology of cholera, although the succession of *V. cholerae* O1 in Mexico remains a mystery, our observations clearly do not support the hypothesis of global transmission of cholera from Africa to Latin America, as proposed elsewhere (8). During the 1990s’ Latin American epidemic, Peru was the first country to have been affected by cholera, and a clonal CTX^+^ *V. cholerae* O1 El Tor strain was found to be the etiological agent, which was present on the Peruvian coast for at least several months prior to the onset of the cholera epidemic (21). Furthermore, CTX^−^ *V. cholerae* O1 El Tor had been isolated from two patients with diarrhea in Lima, Peru, in 1988 (21, 46) and from sewage in Brazil in 1982 (21, 47). The environmental stimulus for *V. cholerae* (i.e., the increase in the temperature and phytoplankton abundance due to the El Nino phenomenon or changes in salinity and/or nutrient concentrations) may have triggered the existing CTX^+^ *V. cholerae* O1 El Tor strains to upsurge rapidly during the 1990s in Peru (21). Molecular typing and phylogenetic analysis of 1990s’ Latin American *V. cholerae* O1 isolates have been done in several studies, and no significant correlation was found between isolates from Asia and Latin America (8, 19). Phylogenetic analysis of the isolates shows that cholera in Mexico during 1991 to 2008 was caused by genetically diverse *V. cholerae* O1 strains belonging to distinct phylogenetic clades. Although, Mexican hybrid isolates show close relatedness to one hybrid isolate from Bangladesh, all of which were isolated in 1991, we do not have sufficient metadata to find out the direction of transmission either from Asia to Latin America or vice versa. Additionally, the antibiotic susceptibility patterns and CTX arrangements of the Mexican isolates strongly contradict the notion of a single-source transmission of *V. cholerae* O1 into Mexico from African countries. The lack of the SXT/R391 ICE in the Latin American CTX^+^ *V. cholerae* isolates is yet another interesting observation, which requires further study, since concurrent Asian and African isolates generally possess SXT/R391 ICE. Therefore, genetic events occurring in *V. cholerae* O1 strains associated with endemic cholera in Mexico are different from those of Asian and African countries (5, 7). Results provided in this study are concordant with those of previous investigations (5, 7, 22) and suggest a likely association of indigenous populations of *V. cholerae* that play a significant role in the dynamics of cholera in Mexico.

## MATERIALS AND METHODS

### Bacterial strains.

*Vibrio cholerae* O1 strains analyzed in the present study (*n* = 6) are listed in Table S1 in the supplemental material with the source, location, and year of isolation. *Vibrio cholerae* O1 strains were provided by the Department of Public Health, Faculty of Medicine, National Autonomous University of Mexico (UNAM) and Centro de Investigación Científica y de Educación Superior de Ensenada. The strains were isolated from cholera patients as part of a nationwide cholera surveillance program conducted between 1983 and 2008 in Mexico (5, 7). The bacterial strains were shipped in T1N1 soft agar (1% trypticase, 1% NaCl, 0.7% agar [pH 7.4]), and the identities were confirmed by standard culture methods and biochemical tests, followed by serogroup and biotype determination, as described previously (48, 49).

### Sequencing, assembly, and annotation.

Genomic DNA of six *V. cholerae* strains was subjected to next-generation whole-genome Illumina and hybrid Illumina/454 sequencing and closure strategies, as previously described (11, 14). Libraries were constructed with target insert sizes of 3 kb and paired-end sizes of 100 bp. Hybrid and Illumina sequences were assembled using Celera and Velvet assemblers, respectively (50) and all chromosomes were manually annotated using the Manatee system (http://manatee.sourceforge.net/).

### Comparative genomics.

Genome-to-genome comparison was performed by using different approaches because the completeness and quality of the nucleotide sequences varied from strain to strain. First, ORFs of a given pair of genomes were identified and reciprocally compared with each other using the BLASTN, BLASTP, and tBLASTx programs (ORF-dependent comparison). Second, a bioinformatic pipeline was constructed to identify homologous regions of a given query ORF. Initially, a segment on the target contig, which is homologous to a query ORF, was identified using the BLASTN program. This potentially homologous region was expanded in both directions by 2,000 bp. Nucleotide sequences of the query ORF and selected target homologous regions were aligned using a pairwise global alignment algorithm, and the resultant matched region in the subject contig was extracted and saved as a homologue (ORF-independent comparison). Orthologues and paralogs were differentiated by reciprocal comparison, as described previously (11).

### Identification and annotation of genomic islands.

We defined genomic islands (GIs) as a continuous array of five or more coding sequences (CDSs) that were discontinuously distributed among genomes of test strains. Correct transfer or insertion of GIs was readily differentiated from a deletion event by comparing the genome-based phylogenetic tree and full matrices showing pairwise detection of orthologous genes between test strains. Identified GIs were designated and annotated using the BLASTP search of its member CDSs against the GenBank NR database, as described elsewhere (11).

### Phylogenetics based on genome sequences.

Orthologous regions of *V. cholerae* N16961 were identified by comparisons based on similarity and were used to generate phylogenetic trees (14). The set of orthologous regions for each CDS of a reference genome was identified according to nucleotide similarity and aligned using CLUSTALW2. The resultant multiple alignments were concatenated to form genome-scale alignments, which were then used to generate the neighbor-joining phylogenetic trees (51).

### Nucleotide sequence accession numbers.

Whole-genome sequences of CP1030, CP1032, CP1033, CP1035, CP1037, and 95412 have been deposited in the DDBJ/EMBL/GenBank databases under accession no. ALCZ00000000, ALDA00000000, AJRL00000000, AJRM00000000, ALDB00000000, and APFM00000000, respectively.

## SUPPLEMENTAL MATERIAL

Table S1  Phenotypic and genotypic characteristics of *V. cholerae* O1 strains (*n* = 182) isolated from clinical and environmental samples collected in Mexico from 1983 to 2008 (adopted from Alam et al. [5, 7]). Table S1, DOCX file, 0.03 MB

Table S2  Distribution of genomic islands (GIs) in *Vibrio cholerae* O1 strains isolated in Mexico from 1991 to 2008. Table S2, DOCX file, 0.03 MB
