# Supplementary material for: Phylogenetic Diversity of Vibrio cholerae Associated with Endemic Cholera in Mexico from 1991 to 2008
Source: mBio. 2016 Mar 15;7(2):e02160-15. doi: 10.1128/mBio.02160-15 (PMC4807371; doi:10.1128/mBio.02160-15)
Supplement: Table S1 — Phenotypic and genotypic characteristics of V. cholerae O1 strains (n = 182) isolated from clinical and environmental samples collected in Mexico from 1983 to 2008 (adopted from Alam et al. [5, 7]). [file mbo002162737st1.docx]

**SI Table 1**. Phenotypic and genotypic characteristics of *V. cholerae* O1 strains (n = 182) isolated from clinical and environmental samples collected in Mexico (1983 - 2008) (adopted from Alam, *et al*., 2010 and Alam, *et al*., 2014).

| **Year of isolation** | **No. of  strains** | **Source** | **Serotype** | **Phenotypic properties** | | |  | **Genetic screening by PCR** | | | | | | | **Deduced  biotype** |
| --- | --- | --- | --- | --- | --- | --- | --- | --- | --- | --- | --- | --- | --- | --- | --- |
|  |  |  |  | **Poly B  (50U)** | **Phage  IV (CL)** | **Phage  V (ET)** |  | ***ompW*** | ***wbeO1*** | ***ctxA*** | ***ctxB* type** | ***tcpA*** | ***rtxC*** | ***rstR* type** |  |
| 1983 | 1 | Human | Inaba | S | S | R |  | + | + | + | CL | CL | - | CL | CL |
| 1991 | 21* | Human | Ogawa | R | R | S |  | + | + | + | CL | ET | ET | CL, ET | Alt-ET* |
|  | 2 | Human | Ogawa | S | R | S |  | + | + | + | CL | ET | ET | CL, ET | Alt-ET |
|  | 6 | Human | Inaba | R | R | S |  | + | + | + | ET | ET | ET | ET | ET |
| 1992 | 3 | Human | Inaba | R | R | S |  | + | + | + | ET | ET | ET | ET | ET |
|  | 1 | Env. | Inaba | S | R | S |  | + | + | + | ET | ET | ET | ET | ET |
|  | 1 | Human | Inaba | R | R | R |  | + | + | + | ET | ET | ET | ET | ET |
|  | 1 | Env. | Inaba | S | R | R |  | + | + | - | - | CL | - | CL | CL |
| 1993 | 6 | Human | Ogawa | R | R | S |  | + | + | + | CL | ET | ET | CL, ET | Alt-ET |
|  | 1 | Human | Ogawa | S | R | S |  | + | + | + | ET | ET | ET |  | ET |
|  | 1 | Human | Ogawa | S | R | S |  | + | + | + | CL | ET | ET | CL, ET | Alt-ET |
| 1994 | 1 | Human | Inaba | R | R | S |  | + | + | + | ET | ET | ET |  | ET |
|  | 1 | Env. | Inaba | R | R | S |  | + | + | + | CL | ET | ET | CL, ET | Alt-ET |
| 1995 | 2 | Human | Ogawa | R | R | S |  | + | + | + | CL | ET | ET | CL, ET | Alt-ET |
|  | 1 | Human | Ogawa | S | R | R |  | + | + | + | CL | CL | - | CL | CL |
|  | 1 | Env. | Inaba | R | R | R |  | + | + | + | CL | CL | - | CL | CL |
|  | 1 | Human | Inaba | R | R | S |  | + | + | + | ET | ET | ET | ET | ET |
|  | 1 | Human | Inaba | R | R | S |  | + | + | + | CL | ET | ET | CL, ET | Alt-ET |
|  | 1 | Env. | Ogawa | R | R | R |  | + | + | + | CL | ET | ET | CL, ET | Alt-ET |
|  | 1 | Human | Inaba | S | R | S |  | + | + | + | CL | ET | ET | CL, ET | Alt-ET |
| 1997 | 34 | Human | Ogawa | R | R | S |  | + | + | + | CL | ET | ET | CL, ET | Alt-ET |
|  | 1 | Env. | Inaba | R | R | S |  | + | + | + | CL | ET | ET | CL, ET | Alt-ET |
|  | 1 | Env. | Inaba | S | S | R |  | + | + | + | CL | CL | - | CL | CL |
|  | 1* | Human | Inaba | S | S | R |  | + | + | + | CL | CL | - | CL | CL* |
| 1998 | 3 | Human | Ogawa | R | R | S |  | + | + | + | CL | ET | ET | CL, ET | Alt-ET |
| 1999 | 4 | Human | Ogawa | R | R | S |  | + | + | + | CL | ET | ET | CL, ET | Alt-ET |
|  | 5 | Human | Ogawa | R | R | S |  | + | + | - | - | ET | ET | - | ET |
|  | 1 | Human | Ogawa | R | R | R |  | + | + | - | - | ET | ET | - | ET |
|  | 1 | Human | Inaba | R | R | S |  | + | + | - | - | ET | ET | - | ET |
| 2000 | 8* | Human | Ogawa | R | R | S |  | + | + | + | CL | ET | ET | CL, ET | Alt-ET* |
|  | 1 | Human | Ogawa | R | R | R |  | + | + | + | CL | ET | ET | CL, ET | Alt-ET |
|  | 2 | Human | Ogawa | R | R | S |  | + | + | + | ET | ET | ET | ET | ET |
|  | 4 | Env. | Inaba | R | R | S |  | + | + | - | - | - | ET | - | ET |
|  | 1 | Human | Ogawa | R | R | S |  | + | + | - | - | ET | ET | - | ET |
| 2001 | 3 | Env. | Ogawa | R | R | R |  | + | + | - | - | - | ET | - | ET |
|  | 2 | Env. | Ogawa | R | R | S |  | + | + | - | - | ET | ET | - | ET |
|  | 2 | Env. | Ogawa | R | R | S |  | + | + | - | - | CL | ET | - | TCP-Var |
|  | 1 | Human | Ogawa | R | R | S |  | + | + | - | - | ET | ET | - | ET |
|  | 1 | Env. | Inaba | R | R | R |  | + | + | - | - | - | ET | - | ET |
| 2002 | 1 | Human | Ogawa | R | R | S |  | + | + | - | - | - | ET | - | ET |
|  | 1 | Human | Ogawa | R | R | S |  | + | + | - | - | ET | ET | - | ET |
|  | 1 | Env. | Inaba | R | R | S |  | + | + | - | - | ET | ET | - | ET |
| 2003 | 1 | Human | Ogawa | R | R | S |  | + | + | - | - | - | ET | - | ET |
|  | 1 | Env. | Ogawa | R | R | S |  | + | + | - | - | - | ET | - | ET |
|  | 1 | Env. | Inaba | R | R | S |  | + | + | - | - | - | ET | - | ET |
|  | 1* | Env. | Ogawa | R | R | S |  | + | + | - | - | CL | ET | - | TCP-Var* |
| 2004 | 4 | Env. | Inaba | R | R | S |  | + | + | + | + | ET | ET | ET | ET |
|  | 3 | Env. | Ogawa | R | R | S |  | + | + | - | - | ET | ET | - | ET |
|  | 1 | Human | Ogawa | R | R | S |  | + | + | - | - | CL | ET | - | TCP-Var |
|  | 1* | Human | Ogawa | R | R | R |  | + | + | - | - | - | ET | - | ET* |
| 2005 | 10 | Env. | Inaba | R | R | S |  | + | + | + | + | ET | ET | ET | ET |
| 2006 | 11 | Env. | Inaba | R | R | S |  | + | + | + | + | ET | ET | ET | ET |
| 2007 | 3 | Human | Inaba | R | R | R |  | + | + | + | + | ET | ET | ET | ET |
|  | 2 | Env. | Inaba | R | R | S |  | + | + | + | + | ET | ET | ET | ET |
|  | 1 | Human | Inaba | R | R | S |  | + | + | + | + | ET | ET | ET | ET |
| 2008 | 8* | Env. | Inaba | R | R | S |  | + | + | + | + | ET | ET | ET | ET* |
|  | 1 | Env. | Inaba | R | R | R |  | + | + | + | + | ET | ET | ET | ET |
| N19961 (Reference) | | Human | Inaba | R | R | S |  | + | + | + | + | ET | ET | ET | El Tor |
| O395 (Reference) | | Human | Ogawa | S | S | R |  | + | + | + | + | CL | CL | CL | Classical |
| Alt, altered; CL, classical; ET, El Tor; Env., environment; Poly B, polymixin B; R, resistant; S, sensitive; TCP-Var, TCP-variant El Tor;  +, positive; -, negative. *Whole genome sequencing has been performed of one representative strain of the genotype | | | | | | | | | | | | | | | |
