# Supplementary material for: Phylogenetic Diversity of Vibrio cholerae Associated with Endemic Cholera in Mexico from 1991 to 2008
Source: mBio. 2016 Mar 15;7(2):e02160-15. doi: 10.1128/mBio.02160-15 (PMC4807371; doi:10.1128/mBio.02160-15)
Supplement: Table S2 — Distribution of genomic islands (GIs) in Vibrio cholerae O1 strains isolated in Mexico from 1991 to 2008. [file mbo002162737st2.docx]

**SI Table 2.** Distribution of genomic islands (GIs) in *Vibrio cholerae* O1 strains isolated in Mexico (1991-2008).

| **Genomic island  (GI)** | **insert site** | **Chr** | **N16961** | **INDRE 91/1** | **IEC224** | **CP1030** | **CP1032** | **CP1033** | **O395** | **95412** | **CP1037** | **CP1035** | **IS/transposase/integrase** | **pathogenicity** | **restriction- modification system** |
| --- | --- | --- | --- | --- | --- | --- | --- | --- | --- | --- | --- | --- | --- | --- | --- |
| VSP-1 | VC0174 - VC0186 | 1 | + | + | + | + | + | + | - | - | - | - | -/transposase, putative/phage Integrase | Vibrio seventh pandemic island-1 | - |
| VSP-2 | VC0489 - VC0517 | 1 | + | + | + | + | + | + | - | - | - | - | -/transposaseOrfAB,transposases/phage integrase | Vibrio seventh pandemic island-2 | - |
| VPI-1 | VC0809 - VC0848 | 1 | + | + | + | + | + | + | + | + | + | - | -/transposases/phage integrase | Vibrio pathogenicity island-1; Cytoplasmic, CoA-independent, aldehyde dehydrogenase (EC 1.2.1.3)/Lipoprotein, ToxR-activated gene, TagA/TCP/AcfABCD/TagE | - |
| VPI-2 | VC1757 - VC1810 | 1 | + | + | + | + | + | + | + | + | - | - | -/transposaseOrfAB/phage integrases | Vibrio pathogenicity island-2: chemotaxis protein MotB-related protein/Sialic Acid Metabolism | Type I |
| CTX | VC1451 - VC1465/VCA0569 - VCA0570 | 1/2 | + | + | + | + | + | + | + | + | - | - | - | Cholera toxin, Zonaoccludens toxin, Accessory cholera enterotoxin, colonization factor | - |
| RS1 | VC1451 - VC1465 | 1 | + | + | + | - | + | + | - | - | - | - | - | - | - |
| TLC | VC1464~VC1479 | 1 | + | + | + | + | + | + | + | + | - | - | -/transposaseOrfAB/- | Cryptic plasmid linked to the CTX prophage | - |
| GI-01 | VC1393 - VC1405 | 1 | + | + | + | + | + | + | + | + | + | - | - | Bacterial Chemotaxis; CheABWRY | - |
| GI-02 | VC1582 - VC1588 | 1 | + | + | + | + | + | + | + | + | + | - | - | - | - |
| GI-03 | VC1747 - VC1753 | 1 | + | + | + | + | + | + | + | + | + | - | - | - | - |
| GI-04 | VC1819 - VC1828 | 1 | + | + | + | + | + | + | + | + | + | - | - | - | - |
| GI-05 | VCA0197 - VCA0204 | 2 | + | + | + | + | + | + | + | + | - | - | IS1004 transposase/- | - | - |
| GI-06 | VCA0280b - VCA0287 | 2 | + | + | + | + | + | + | + | + | - | - | IS5 transposase/phage Integrase | Uropathogenic specific protein | - |
| GI-07 | VCA0666 - VCA0674a | 2 | + | + | + | + | + | + | + | + | + | - | - | - | - |
| GI-08 | VCA0789 - VCA0796 | 2 | + | + | + | + | + | + | + | + | + | - | -/transposaseOrfAB/possible integrase | - | - |
| GI-09 | VCA0849 - VCA0858 | 2 | + | + | + | + | + | + | + | + | + | - | - | - | - |
| GI-10 | VCA0877 - VCA0885 | 2 | + | + | + | + | + | + | + | + | - | + | -/-/integrase-related protein | Non-hemolytic enterotoxin lytic component L1 | - |
| GI-11‡ | VCA0197 | 2 | - | - | - | - | - | - | + | + | - | - | -/-/Integrase | - | - |
| GI-14 | VCA0480 - VCA0481 | 2 | - | - | - | - | - | - | + | +* | - | - | - | - | - |
| GI-15 | VC0002 - VC0003 | 1 | - | - | - | - | - | + | - | - | - | - | -/-/integrases, putative integrase | Dihydropteroate synthase; Streptomycin 3''-O-adenylyltransferase (EC 2.7.7.47) | - |
| GI-17 | VCA0885 | 2 | - | - | - | - | - | - | - | - | + | - | -/-/integrase-related protein | Streptococcal hemagglutinin protein, Methyl-accepting chemotaxis proteins | - |
| GI-19 | VCA0569 - VCA0570 | 2 | - | - | - | - | - | - | - | - | +* | - | -/-/phage integrase / RstB phage-related integrase | Zona occludens toxin, Accessory cholera enterotoxin | - |
| GI-23 | VC0154 - VC0156 | 1 | - | - | - | - | - | - | + | + | - | - | -/transposases, transposaseOrfAB/site-specific recombinase,  phage integrase family | - | - |
| GI-24 | VC0289 - VC0290 | 1 | - | - | - | - | - | - | + | + | - | - | -/-/site-specific recombinase, phage integrase family | CRISPR-associated proteins | - |
| GI-34 | VC2714 - VC2715 | 1 | - | - | - | - | - | - | - | - | - | + | - | - | - |
| GI-35 | VC1910 - VC1911 | 1 | - | - | - | - | - | - | - | - | - | + | - | - | - |
| GI-36 | VC2041 - VC2042 | 1 | - | - | - | - | - | - | - | - | + | - | -/-/integrase | - | - |
| GI-40 | VCA0607 - VCA0608 | 2 | - | - | - | - | - | - | - | - | - | + | - | - | - |
| GI-47 | VC0809 - VC0848 | 1 | - | - | - | - | - | - | - | - | + | - | - | - | - |
| GI-74¥ | VC1494 | 1 | - | + | + | + | - | - | - | - | - | - | - | - | - |
| GI-85 | VC1854 - VC1835 | 1 | + | - | + | + | + | + | + | + | + | + | - | - | - |
| GI-100 | VCA0150 - VCA0151 | 2 | - | - | - | - | - | - | - | - | - | + | - | - | - |
| GI-106 | VC1451 - VC1479 | 1 | - | - | - | - | - | - | - | - | - | + | - | - | - |
| GI-112 | VC0217 | 1 | - | - | - | - | - | - | - | - | + | - | - | - | - |
| GI-125 | VC0002 - VC0003 | 1 | - | - | - | - | - | - | - | - | - | + | -/-/integrase | - | Type I |
| GI-126 | VC0807 - VC0848 | 1 | - | - | - | - | - | - | - | - | - | + | -/-/phage integrase | - | Type I |
| GI-127 | VCA0197 - VCA0204 | 2 | - | - | - | - | - | - | - | - | - | + | - | - | - |
| GI-151 | VC0208 - VC0209 | 1 | - | - | - | - | - | + | - | - | - | - | -/-/integrase | part of VSP-2, 2 bacteriocin immunity proteins | - |
| Chr, chromosome; * position; not determined clearly; ‡ Kappa prophage; ¥ genomic island WASA1 (West African-South American 1) | | | | | | | | | | | | | | | |
